# The roles of mutated SWI/SNF complexes in the initiation and development of hepatocellular carcinoma and its regulatory effect on the immune system: A review

**DOI:** 10.1111/cpr.12791

**Published:** 2020-03-11

**Authors:** Bo Hu, Jian‐Zhen Lin, Xiao‐Bo Yang, Xin‐Ting Sang

**Affiliations:** ^1^ Department of Liver Surgery Peking Union Medical College Hospital Chinese Academy of Medical Sciences and Peking Union Medical College Beijing China

**Keywords:** chromatin remodelling, hepatocellular carcinoma, immunotherapy, SWI/SNF complex, tumour suppressor gene

## Abstract

Hepatocellular carcinoma (HCC) is a primary liver malignancy with a high global prevalence and a dismal prognosis. Studies are urgently needed to examine the molecular pathogenesis and biological characteristics of HCC. Chromatin remodelling, an integral component of the DNA damage response, protects against DNA damage‐induced genome instability and tumorigenesis by triggering the signalling events that activate the interconnected DNA repair pathways. The SWI/SNF complexes are one of the most extensively investigated adenosine triphosphate‐dependent chromatin remodelling complexes, and mutations in genes encoding SWI/SNF subunits are frequently observed in various human cancers, including HCC. The mutated SWI/SNF complex subunits exert dual functions by accelerating or inhibiting HCC initiation and progression. Furthermore, the abnormal SWI/SNF complexes influence the transcription of interferon‐stimulated genes, as well as the differentiation, activation and recruitment of several immune cell types. In addition, they exhibit synergistic effects with immune checkpoint inhibitors in the treatment of diverse tumour types. Therefore, understanding the mutations and deficiencies of the SMI/SNF complexes, together with the associated functional mechanisms, may provide a novel strategy to treat HCC through targeting the related genes or modulating the tumour microenvironment.

## 1 BACKGROUND

Hepatocellular carcinoma (HCC) is the second leading cause of cancer‐related deaths worldwide, afflicting approximately 800 000 people annually.^1^ The pathogenesis of HCC is extremely complicated, involving processes such as cell cycle regulation and signal transduction, and it reflects the functions and interactions of multiple genes at multiple steps.^2^ HCC is currently treated by surgical resection and chemotherapy, but the mortality rate of this cancer remains high. In recent years, the use of immune checkpoint inhibitors (ICIs), such as ipilimumab (the inhibitor of cytotoxic T‐lymphocyte antigen 4 [CTLA4]) and nivolumab (the inhibitor of programmed death‐1 [PD‐1]), have demonstrated survival benefits for HCC, which reveals that immune status is closely related to HCC progression.^3^, ^4^ Therefore, it is important to further explore the characteristics of HCC and to develop novel therapies for treating this cancer.

**FIGURE 1:**
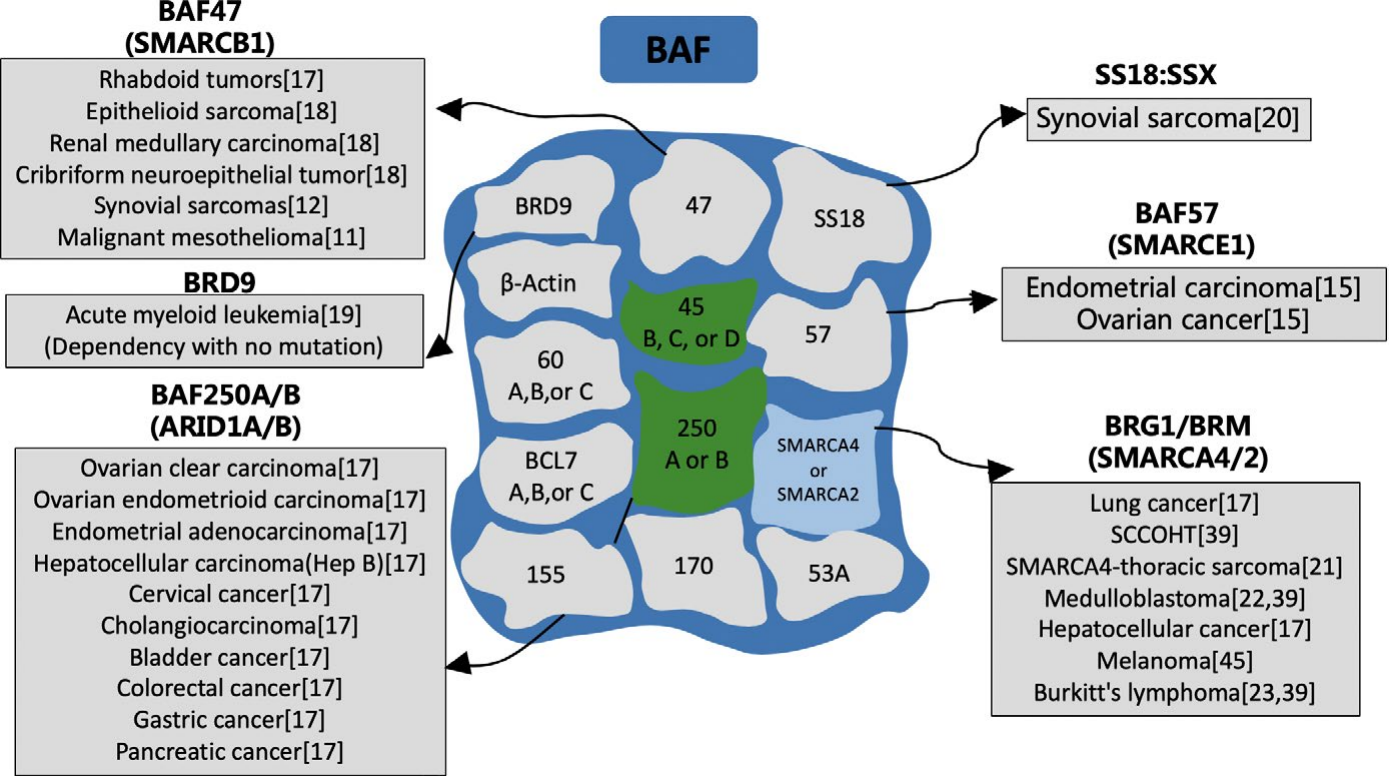
High‐frequency gene mutation of BAF in different tumours. The sources are shown in brackets. BAF, BRG1‐associated factor

**FIGURE 2:**
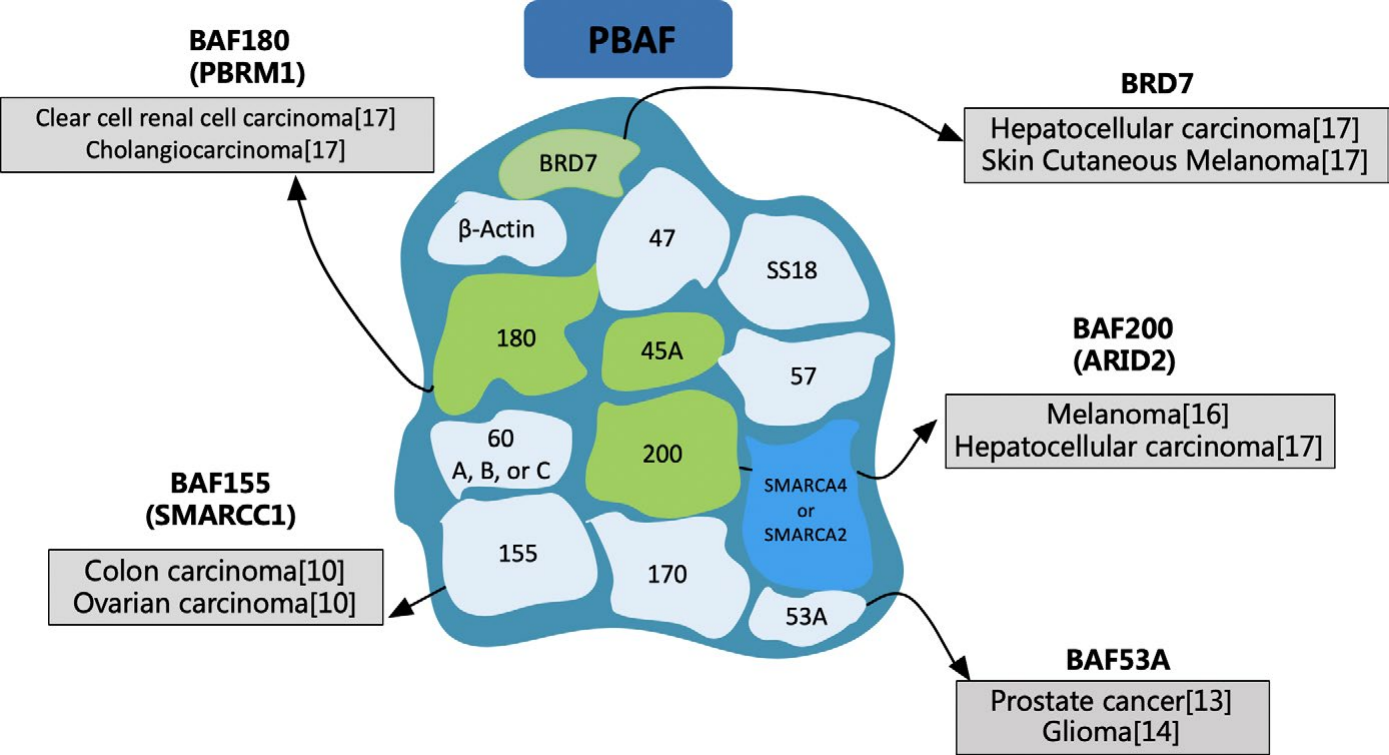
High‐frequency gene mutation of PBAF in different tumours. The sources are shown in brackets. PBAF, polybromo BAF

The mating‐type switch/sucrose non‐fermenting (SWI/SNF) complexes, which are capable of regulating gene transcription through adenosine triphosphate (ATP)‐dependent nucleosome remodelling, have been shown to play a widespread role in carcinogenesis.^5^ The SWI/SNF complexes are macromolecular complexes comprising 12‐15 subunits, including a catalytic ATPase subunit, SWI/SNF related, matrix associated, actin‐dependent regulator of chromatin, subfamily a, member 4 (SMARCA4)/brahma‐related gene 1 (BRG1) or SMARCA2/brahma (BRM); and several core subunits, such as SMARCB1/SNF5/INI1/BAF47 or SMARCC1/BAF155. Other subunits, such as AT‐rich interaction domain 1A (ARID1A) and ARID1B, are the mutually exclusive components of the BRG1‐associated factor (BAF) complexes, while polybromo 1 (PBRM1) and ARID2 are specific for the polybromo BAF (PBAF) complexes.^6^ Recently, a third SWI/SNF complex called non‐canonical BAF (ncBAF, also termed GBAF) has been identified, which contains glioma tumour suppressor candidate region gene 1 (GLTSCR1)/GLTSCR1‐like (GLTSCR1L) subunits instead of the ARID‐domain containing proteins.^7^ Global genomic analyses suggest that the SWI/SNF complexes are associated with a mutation rate of 20% among all human tumours.^8^ Frequent inactivating mutations in SWI/SNF subunits, such as ARID1A, ARID1B, ARID2, PBRM1 and SMARCA4, are repeatedly detected in numerous cancers (Figures 1 and 2).^9^, ^10^, ^11^, ^12^, ^13^, ^14^, ^15^, ^16^, ^17^, ^18^, ^19^, ^20^, ^21^, ^22^, ^23^ The mutation proportion and types of different SWI/SNF complex subunits in several HCC data sets are summarized in Table 1. In addition, increasing evidence indicates that the SWI/SNF complexes directly interact with numerous important proteins, such as beta‐catenin, to modulate cancer formation.^24^ The SWI/SNF complexes not only exert a vital part in transcription activation but also participate in transcriptional repression.^25^ Furthermore, an increasing number of studies reveal that the ATP‐dependent chromatin remodelling complexes, like SWI/SNF, exhibit significant impacts on human immunodeficiency virus type 1 (HIV‐1),^26^ autoimmune reactivity^27^ and hypersensitivity.^28^ Nonetheless, the role of SWI/SNF complexes in HCC occurrence and development as well as the immune system has not yet been fully elucidated. This review integrates the available data on the role of aberrant SWI/SNF complexes during HCC progression and examines its regulatory effects on several immune cell types and ICI therapies.

**TABLE 1:** The proportion and types of SWI/SNF mutations in different hepatocellular carcinoma data sets

Gene	Proportion (%)	Mutation type
ARID1A	9.0	Truncating mutation, missense mutation, fusion, inframe mutation, amplification, deep deletion
ARID2	5.0	Truncating mutation, missense mutation, inframe mutation, deep deletion
ARID1B	2.9	Truncating mutation, missense mutation, amplification, deep deletion
SMARCA4	2.5	Truncating mutation, missense mutation, fusion, amplification, deep deletion
BRD9	2.3	Truncating mutation, missense mutation, amplification
SMARCA2	2.1	Truncating mutation, missense mutation, amplification, deep deletion
PHF10	2.1	Truncating mutation, missense mutation, amplification, deep deletion
PBRM1	2.1	Truncating mutation, missense mutation, fusion, amplification, deep deletion
BRD7	1.8	Truncating mutation, missense mutation, deep deletion
SMARCC2	1.6	Truncating mutation, missense mutation, fusion, amplification, deep deletion
BCL11B	1.4	Truncating mutation, missense mutation, deep deletion
SMARCD2	1.3	Missense mutation, amplification
SMARCE1	0.9	Missense mutation, amplification, deep deletion
SMARCB1	0.9	Missense mutation, amplification, deep deletion
ACTL6A	0.8	Truncating mutation, missense mutation, amplification
BCL7B	0.8	Missense mutation, fusion, amplification
SMARCC1	0.6	Truncating mutation, missense mutation, deep deletion
SMARCC3	0.4	Missense mutation, amplification, deep deletion
SS18	0.2	Missense mutation, amplification
BCL7A	0.1	Missense mutation
BCL7C	0.1	Amplification

Abbreviation: NA, non‐available.

## 2 ABERRANT BAF COMPLEXES PLAY DIVERSE ROLES IN HCC DEVELOPMENT

In mammals, BAF complexes are composed of a single central ATPase, either SMARCA4/BRG1 or SMARCA2/BRM, and several BRG‐/BRM‐associated factors.^29^ In addition to the subunits homologous to those in Drosophila or yeast, several other subunits appear to be dedicated to vertebrate or mammalian complexes, including SS18/SS18L1, BCL7A/B/C and BCL11/A/B. BAF complexes have been implicated in embryonic development.^30^ The presence of the BAF complex on chromatin correlates with that of enhancers,^31^ and its activity regulates a variety of important biological processes ranging from self‐renewal and pluripotency in embryonic stem cells^32^ to cardiac development.^33^ A previous study revealed that cancers with a perturbation in BAF components were dependent on ncBAF,^7^ highlighting a synthetic lethal relationship between the two distinct SWI/SNF complexes as opposed to paralogs of the same complex. Research on the role of ncBAF in the development of HCC is still rare, although BAF subunits are frequently disrupted in a variety of different malignancies including HCC.

### 2.1 *SMARCA4*/*BRG1*

*SMARCA4* is located in chromosome 19p13.2, and its protein functions as the central catalytic component in the SWI/SNF complex. Specifically, the SMARCA4 protein comprises multiple domains, including an evolutionarily conserved catalytic ATPase domain, a conserved C‐terminal bromodomain, AT‐hook motif and the less characterized N‐terminal region, all of which play critical roles in modified histone protein recognition, DNA binding or SWI/ SNF recruitment.^34^, ^35^, ^36^, ^37^ SMARCA4 has been demonstrated to interact with diverse nuclear proteins involved in various cellular processes, such as transcriptional regulation, cell cycle control, proliferation, DNA repair and recombination.^38^ However, the role of SMARCA4 in cancer occurrence and progression remains unclear. It is worth noting that *SMARCA4* has been proposed to act as a tumour suppressor gene through diverse biological mechanisms, since frequent inactivating mutations are detected in various tumour cell lines, such as lung, small‐cell carcinoma of the ovary (SCCOHT), hypercalcemic type, medulloblastoma and Burkitt's lymphoma.^39^ Firstly, reintroducing SMARCA4 into *SMARCA4*‐deficient tumour cells resulted in Rb‐dependent cell cycle arrest and a flattened morphology, suggesting that *SMARCA4* may function as a tumour suppressor gene.^40^, ^41^ Furthermore, *SMARCA4* heterozygotes were predisposed to the development of differentiated epithelial tumours, which suggests that SMARCA4 plays a vital part in regulating cell proliferation.^42^ In addition, *SMARCA4* was shown to participate in tumour suppression based on interactions with other tumour suppressor genes, such as retinoblastoma protein (*pRb*), *p53* and *c‐Myc*.^43^, ^44^ Paradoxically, *SMARCA4* is also reported to be mutated and over‐expressed in a variety of malignant tumours, including melanoma, gastric and prostate cancers. As a result, it induces tumour proliferation by regulating the expression of cyclin D1 and cyclin E, driving the cells to enter S phase in the cell cycle and displaying a marked correlation with poor survival.^45^, ^46^, ^47^, ^48^ Indeed, it has been suggested that SMARCA4 directly up‐regulates enzymes responsible for fatty acid (FA) and lipid biosynthesis, which can be utilized by cancer cells to provide energy for proliferation.^49^ Other underlying mechanisms by which SMARCA4 promotes the proliferation of different tumour cells include stimulating the expression of *MYC*, inhibiting that of phosphatase and tensin homolog (*PTEN*), further reducing the active components in the phosphatidylinositol‐3 kinase‐protein kinase B (PI3K‐AKT) signalling pathway and promoting the function of the WNT signalling pathway.^50^, ^51^, ^52^ Such a confusing situation may be due to the variety of SMARCA4 functions depending on different tumour types or even the different stages and subtypes of the same tumour.

In the context of HCC, Zhong et al^53^ showed that the *SMARCA4* single nucleotide polymorphism rs11879293, which was located in the intron between exon 1 and exon 2 of *SMARCA4* and was essential for the interaction between SMARCA4 and SS18L1/CREST, showed a remarkable association with a reduced risk of HCC, as verified in stage 2 combined analysis. The mechanism underlying this association may be that rs11879293 acts as an intronic enhancer to alter the Pol2‐binding site; alternatively, it tags the functional variants in the promoter/regulatory region that affect gene expression, thus conferring susceptibility to HCC. Furthermore, the authors note that the hepatitis B virus (HBV) infection status resulted in a greatly differentiated risk between *SMARCA4* rs11879293 and HCC incidence. They also speculated that SMARCA4 might possess similar DNA‐binding characteristics with SMARCE1, a core SWI/SNF subunit for modulating the HBV replication efficiency, and in this context, SMARCA4 may also act as a regulator of HBV replication. In contrast, instead of suggesting *SMARCA4* as a tumour suppressor gene, Kaufmann et al^45^ provided evidence that the over‐expression of *SMARCA4* was observed to enhance cell growth and invasiveness of HCC and that it might be linked to cyclin B, cyclin E and matrix metalloproteinase 7 (MMP7). Other studies indicate that up‐regulation of the metastasis‐associated lung adenocarcinoma transcript 1 (MALAT1), the p53 pathway, as well as the lipopolysaccharide (LPS)‐induced pro‐inflammatory mediators IL‐6 and C‐X‐C motif chemokine 8 (CXCL8), may be involved in hepatocarcinogenesis.^54^, ^55^ In more recent studies, when copy number analysis was used in combination with expression profiling to identify cancer‐associated mutations, the results revealed that *SMARCA4* is up‐regulated in HCC and that its level is markedly correlated with cancer progression among HCC patients.^56^ Additionally, the nuclear expression of *SMARCA4* also predicts the early recurrence of HCC in affected patients. Furthermore, SMARCA4, which is found to facilitate S‐phase entry and attenuate apoptosis, also promotes cell proliferation through the up‐regulation of *SMAD6*. On the other hand, it has been shown that SMARCA4 aggravates liver fibrosis through regulating the activation of hepatic stellate cells (HSCs) via the transforming growth factor beta (TGFβ)/SMAD signalling pathway.^57^ Moreover, *Smarca4*‐deficient mice display decreased HSC activation in vitro, along with reduced liver fibrogenesis after chronic damage induced by carbon tetrachloride (CCl_4_) administration. This suggests that SMARCA4 may also be instrumental in the progression of hepatic fibrosis to HCC. The above‐mentioned evidence appears to indicate that *SMARCA4*, previously considered to be a tumour suppressor gene, serves as a cancer promoter in the context of HCC. However, the fundamental role of any given BAF and PBAF alteration in cancer is likely to be unique to each cancer type and may reflect the idiosyncratic processes that drive each malignancy (whether oncogene addiction, autocrine signalling, mutagen exposure, chromosomal instability, etc.).^58^ Although several mechanisms of action have been proposed above, the specific factors influencing the opposing roles of abnormal SMARCA4 among multiple tumours are still unknown. Whole‐genome sequencing and various new techniques to investigate 3D chromatin architecture may offer substantial insight into the full breadth of the effects resulting from SMARCA4 dysfunction, as well as other subunits, and reveal their diverse contributions towards oncogenesis and tumour biology.

### 2.2 *SMARCA2*/*BRM*

**FIGURE 3:**
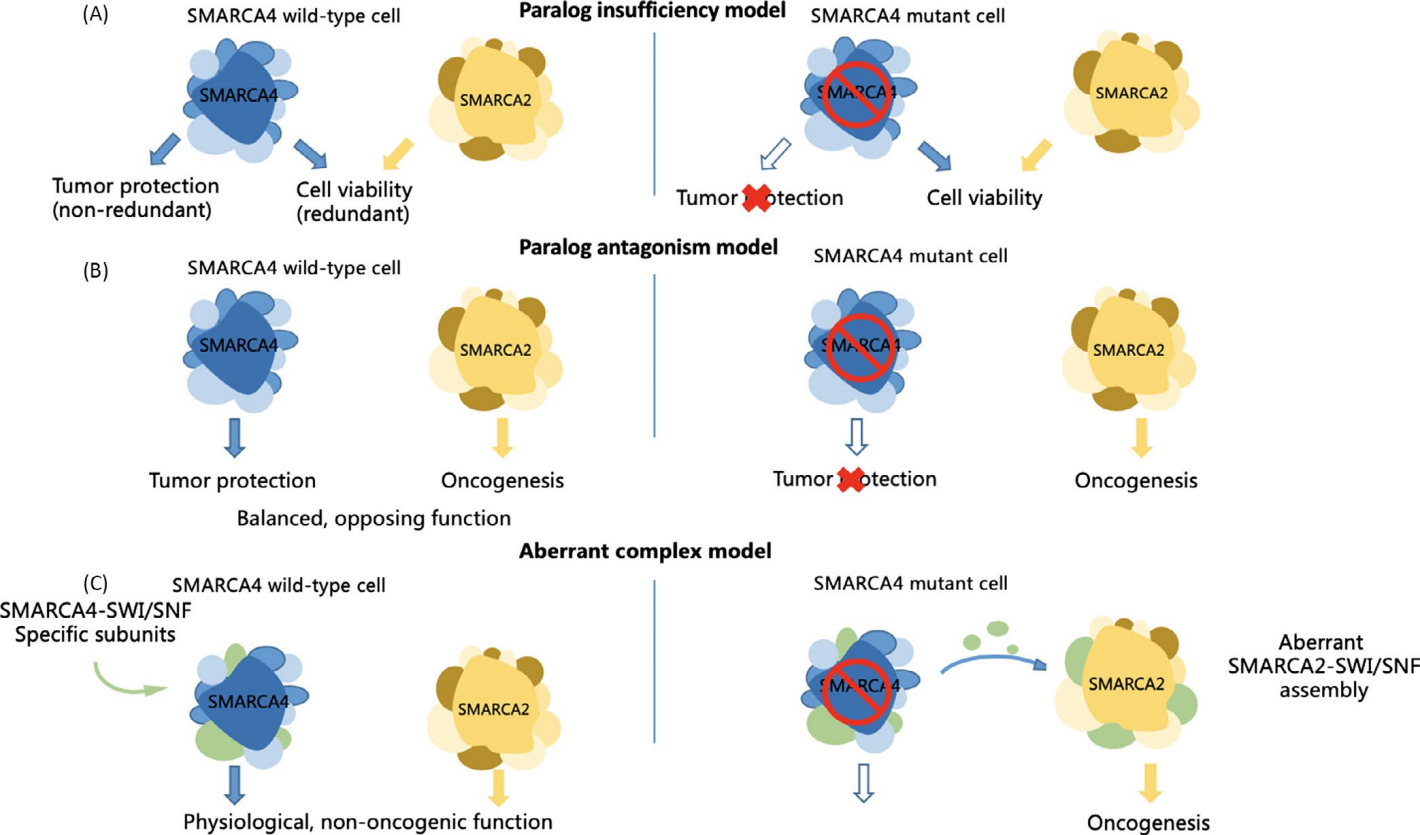
Hypothetical models for the function of residual BRM‐SWI/SNF complexes in BRG1‐mutant cancers. A, Paralog insufficiency model. In the cancer cell of origin, BRG1 and BRM perform redundant functions in supporting cell viability, while BRG1 performs a non‐redundant tumour suppressor function. Loss of BRG1 would lead to tumorigenic effects while simultaneously rendering BRM the sole ATPase subunit responsible for supporting tumour cell viability. B, Paralog antagonism model. In the cancer cell of origin, BRG1 performs a specific function in tumour protection, while BRM promotes oncogenesis, resulting in a balanced state of SWI/SNF functions. Loss of BRG1 would result in unopposed BRM‐driven proliferation and tumorigenesis. C, Aberrant complex model. Loss of BRG1 would release specific subunits of its dedicated protein complex, which would form aberrant associations with BRM that deregulate cancer‐relevant transcriptional programmes

SMARCA2, a homolog with 75% identity to SMARCA4, also regulates chromatin structure, but it is mutually exclusive of SMARCA4 in the SWI/SNF complexes.^59^ SMARCA2 interacts with several transcription factors (TFs) and other DNA‐binding proteins which are involved in chromatin structural modifications that epigenetically regulate gene expression.^60^ *SMARCA2* is not frequently mutated in tumours, but it is silenced in numerous cancer cell lines and primary tumours.^59^ In their computational meta‐analysis, Jose et al^61^ demonstrated that high *SMARCA4* expression was associated with aggressive tumours, while high *SMARCA2* expression was related to benign differentiated tumours, suggesting that SMARCA4 and SMARCA2 play opposite roles in cancers, including HCC. *SMARCA4* and *SMARCA2* also show differential expression patterns during development.^62^ *SMARCA4* tends to be highly expressed in proliferating cells, whereas *SMARCA2* is mainly expressed in cells that cycle slowly (such as stem cells) and non‐cycling differentiated cells.^63^ In contrast, using data from loss‐of‐function screening of 165 cancer cell lines, Boris et al identified that *SMARCA2* was an essential gene in *SMARCA4* mutant cancer cell lines. They note that *SMARCA4* inactivation resulted in greater incorporation of non‐essential SMARCA2 subunits into the SWI/SNF complexes, revealing a role for SMARCA2 in oncogenesis induced by SMARCA4 loss, and they identified that ATPase and bromodomain‐containing SMARCA2 may serve as potential therapeutic targets in these cancers. The hypothetical models depicting the function of residual SMARCA2‐SWI/SNF complexes in *SMARCA4*‐mutant cancers are exhibited in Figure 3. Such findings suggest that the relationship between SMARCA2 and SMARCA4 may be complicated and regulated by different mechanisms; in addition, the specific role of mutated *SMARCA2* in HCC may be transformed due to changes in cancer cell characteristics and the surrounding environment. Nevertheless, more studies investigating the association of SMARCA2 with SMARCA4 and the role of the former in HCC development should be performed.

### 2.3 *SMARCB1*/*BAF47*

SMARCB1, a core subunit of the SWI/SNF complex, has been reported to modulate cell proliferation and apoptosis.^64^, ^65^ The down‐regulation of *SMARCB1*, which is observed in HCC tissues relative to adjacent non‐cancerous tissues, is markedly correlated with poorer overall survival for HCC patients and attenuates the sensitivity of HCC cells to sorafenib.^66^ Additionally, TGF‐β1 expression is shown to be up‐regulated in HCC tissues with reduced *SMARCB1* expression, indicating that SMARCB1 may participate in HCC suppression, and it may serve as an important prognostic biomarker and promising therapeutic target for HCC.

### 2.4 *ARID1A*/*BAF250A*

*ARID1A*, which encodes the BAF250a subunit of the SWI/SNF complexes, is reported to be a tumour suppressor gene that is frequently inactivated by frameshift or truncating mutations, resulting in a lack of protein expression in numerous human cancers, for example ovarian clear cell carcinoma, gastric cancer, HCC, breast cancer, pancreatic cancer, bladder cancer and colon cancer.^67^, ^68^, ^69^, ^70^, ^71^, ^72^, ^73^ As previously stated, the ARID1A‐containing BAF complex remodels the chromatin structure to modulate numerous processes that require DNA access (such as transcription, DNA damage repair and replication) by recruiting and binding to TFs and coactivator/corepressor complexes.^74^ Specifically, ARID1A interacts with topoisomerase IIa (TOP2A), which resolves the sister chromatids linked by the catenated DNA strands during mitosis. *ARID1A* inactivation leads to the activation of the decatenation checkpoint and polyploidy in vitro, and promotes cell cycle progression, cell proliferation, migration, invasion and metastasis.^75^, ^76^ Nevertheless, Zhao et al^77^ demonstrated that, in the case of stromal antigen 1 (*STAG1*) down‐regulation, ARID1A exerted its tumour suppressor function through preserving genomic stability by reducing telomere cohesion; as a result, *ARID1A* inactivation allowed for the elimination of cells with severe genomic instability during mitosis. Consequently, there is a paradoxical phenomenon that, compared with *ARID1A* wild‐type tumours, their *ARID1A*‐mutated counterparts display remarkably less genomic instability, as measured by copy number alteration analysis across multiple cancer types.

When ARID1A mutations were examined for correlations with HCC clinical characteristics, the results suggested that ARID1A alteration is correlated with larger HCC size and well or moderately differentiated HCC, but not with patient age, sex, cirrhosis, tumour node metastasis (TNM) stage, tumour size, number of tumours, vascular invasion, patient survival, HBV infection, hepatitis C virus (HCV) infection, heavy alcohol consumption and diabetes mellitus (Table 2).^78^ Similarly, a study by He et al^79^ suggested that *ARID1A* down‐regulation was markedly associated with HCC tumour growth, poor prognosis and overall metastasis, including local lymph node metastasis and distant metastasis, especially lung metastasis. In addition, the authors also provide evidence that E‐cadherin levels are closely correlated with *ARID1A* expression, suggesting that the latter plays a role in migration and invasion. Typically, the loss of E‐cadherin suppresses E‐cadherin‐dependent cell‐cell adhesion and accelerates cancer development and progression.^80^ Notably, such differences in the correlation of *ARID1A* expression with clinical parameters may be ascribed to the heterogeneous sample sources and statistical criteria. Nevertheless, it is generally accepted that mutated ARID1A partially promotes HCC development. However, the molecular mechanism by which *ARID1A* deficiency or mutation in HCC affects tumour development remains largely unclear. In contrast to the extensively described implications of *ARID1A* as a tumour suppressor gene, Sun et al^81^ demonstrated that *ARID1A* expression played a critical role in initiating HCC, particularly in mediating the liver damage induced by reactive oxygen species through facilitating the transcription of *CYP450* genes (such as Cyp2e1). Intriguingly, in contrast to the initial tumour development stages where ARID1A was shown to support tumorigenesis, the authors also demonstrated that both heterozygous and homozygous *ARID1A* deletions accelerated HCC progression and metastasis following initial tumour establishment. The latter results appear to be consistent with our stated identity of *ARID1A* as a tumour suppressor gene. Loss of function of ARID1A results in HCC angiogenesis through the histone H3‐induced up‐regulation of angiopoietin‐2 (Ang2) via lysine 27 acetylation (H3K27ac) of the Ang2 gene locus.^82^ Alongside these findings, Cheng et al discovered that *ARID1A* was associated with the long non‐coding RNA (lncRNA) *MVIH* among various HCC cell types and hepatoblastoma cells and they identified three interaction domains of *ARID1A* (including amino acids (aa) 311e600, aa 951e1300 and aa 1901e2285). They further suggested that *ARID1A* up‐regulates the expression of cyclin‐dependent kinase inhibitor 1A (*CDKN1A*) and inhibits HCC cell proliferation and migration by suppressing the lncRNA *MVIH*. These findings revealed the potential association of *ARID1A* with lncRNAs and also supported a role for *ARID1A* expression in HCC development.^83^ In addition, the nuclear expression of *p53* or beta‐catenin also showed an inverse correlation with altered *ARID1A* expression, demonstrating that inactivated ARID1A accelerates HCC growth through a pathway that is different from the p53 and beta‐catenin pathways. In their study, Fang et al^84^ show that steatohepatitis, elevated expression of tumour necrosis factor (TNF)‐α and interleukin (IL)‐6, and the activation of the signal transducers and activators of transcription 3 (STAT3) and nuclear factor kappa‐light‐chain‐enhancer of activated B cells (NF‐κB) pathways were detected in liver parenchyma of hepatocyte‐specific *ARID1A* knockout mice, which might contribute to HCC tumorigenesis. Taken together, *ARID1A* expression is required for the initial tumour development and the suppression of HCC metastatic potential, depending on the cellular and temporal contexts during HCC progression. Further in‐depth experiments are needed to explore the HCC microenvironment at different stages that lead to the transformation of ARID1A function, as well as the mechanism of action underpinning this process.

**TABLE 2:** Summary of common SWI/SNF complex mutations and characteristics in hepatocellular carcinoma

Type	Genes	Characters
Hepatocellular carcinoma	ARID1A/BAF250A	Disease characteristics: weak or no ARID1A protein expression is associated with tumour size and differentiation; not associated with age, sex, cirrhosis status, TNM stage, number or tumours, HBV or HCB status, alcohol consumption status, diabetes status and vascular invasion status.
		Survival outcomes: loss of protein expression is not associated with overall and recurrence free survival times in univariate analysis.
	SMARCA2/BRM	Disease characteristics: SMARCA2 mRNA levels are associated with HBV status; not associated with age, sex, tumour size, number of carcinomas, HCV status and disease stage. Loss of BRM protein expression is associated with age, sex, tumour size, HCV status, stage, differentiation and vascular invasion.
		Survival outcomes: loss of protein expression is associated with worse overall survival times in univariate analysis.
	SMARCA4/BRG1	Disease characteristics: SMARCA4 mRNA levels are associated with HCV status; not associated with age, sex, tumour size, number of carcinomas, HBV status and disease stage. Loss of BRG1 protein expression is not associated with age, sex, tumour size, HBV status, HCV status, stage, differentiation and vascular invasion.
		Survival outcomes: loss of protein expression is not associated with overall survival times in univariate analysis.
	ARID2/BAF200	Disease characteristics: ARID2 mRNA levels are associated with age, sex, tumour grade, disease stage and HBV status.
		Survival outcomes: loss of protein expression is not associated with overall survival times in univariate analysis.

Abbreviation: NA, non‐available.

### 2.5 *ARID1B*/*BAF250B*

ARID1B, an isoform that is mutually exclusive with ARID1A in the SWI/SNF complexes and that is involved in regulating transcription and multiple downstream cellular processes, has been recently identified to be the primary mutant gene in various cancers.^85^, ^86^ Previous studies have shown that *ARID1B* gene mutations are responsible for neurodevelopmental retardation, intellectual disability, growth delay and dysmorphic features.^87^ Interestingly, high *ARID1B* expression is associated with poor outcomes in bladder urothelial carcinoma and it also predicts the benefits of adjuvant chemotherapy for this cancer.^86^ Shao et al also suggested that high *ARID1B* expression was tightly correlated with the histological grade and size of invasive breast cancer, and it predicted a reduction in 5‐year disease‐free survival (DFS).^88^ In the context of HCC, Tordella et al^89^ identified that ARID1B serves as a regulator of oncogene‐induced senescence (OIS) and represents a potent tumour suppressor mechanism; they described that knock‐down of *ARID1B* prevented OIS and cooperated with RAS to induce liver tumours. ARID1B not only modulates the transcription of p16 and p21, but also regulates DNA damage, oxidative stress and p53 induction, suggesting that SWI/SNF may employ other mechanisms to regulate senescence. Additionally, the authors also suggest that the expression of ectonucleoside triphosphate phosphohydrolase‐7 (*ENTPD7*) or the inhibition of nucleotide synthesis in *ARID1B*‐depleted cells re‐establishes senescence, shedding more light on the novel mechanisms by which epigenetic regulators affect HCC progression, and suggesting that pro‐senescence therapies can be employed to treat SWI/SNF‐mutated cancers. Using bioinformatic approaches to select three putatively functional variants in *ARID1B* (namely rs73013281C>T, rs167007A>G and rs9397984C>T), Liu et al^90^ revealed that the *ARID1B* variant, rs73013281, made a significant genetic contribution to the susceptibility for HCC, especially for the interaction with physical activity. 

## 3 ALTERATIONS OF PBAF SUBUNIT GENES IN HCC

Polybromo BAF complexes contain PBRM1 and ARID2 but lack ARID1A/B. PBAF complexes also contain BRD7 in place of BRD9,^91^ BAF45A (PHF10) instead of BAF45B/C/D (DPF1/3/2) and lack SS18.^92^ PBAF subunits, which have been shown to be involved in the maintenance of genomic integrity during mitosis,^93^ also regulate cell differentiation and may be an important regulator of cell‐type identity.^94^ Compared with the BAF complexes, PBAF complexes may have distinct or even opposing functional roles. For instance, PBAP but not BAP is required for germinal stem cell maintenance.^95^ On the other hand, visualization of chromatin domains using immunofluorescence shows that BAP and PBAP complexes have both overlapping and mutually exclusive domains. Polycomb domains are not usually found at either of the chromatin domains mentioned above,^96^, ^97^ which suggests that BAP and PBAP work both cooperatively and independently at distinct sites to oppose Polycomb silencing. In HCC, the mutation frequency of PBAF subunit genes is lower than that of BAF subunit genes. Nevertheless, the different roles of the two complexes in the initiation and development of HCC still require further study. The influence of several abnormal PBAF subunit genes on the development of HCC is described in the sections that follow.

### 3.1 *ARID2*/*BAF200*

ARID2 possesses a conservative N‐terminal ARID region, as well as three LLxxLL motifs and two conservative C‐terminal C2H2 Zn‐finger motifs, which directly bind to DNA or interact with proteins.^98^ Recent genomic studies have identified frequent *ARID2* mutations in HCC, but it remains unclear how *ARID2* functions as a tumour suppressor gene. *ARID2* expression is reported to be dramatically down‐regulated in HCC tissues compared with their normal counterparts, and it physically interacts with the E2 promoter binding factor 1 (*E2F1*), decreasing the binding of E2F1/RNA Pol II to the promoters of cyclin D1 (*CCND1*) and cyclin E1 (*CCNE1*). Suppressing ARID2 accelerates the G1/S transition associated with the up‐regulation of *CCND1*, *CCNE1* and cyclin‐dependent kinase 4 (*CDK4*), together with the phosphorylation of the retinoblastoma protein (Rb).^99^ In another study, Oba et al established human *ARID2* knockout HCC cell lines to investigate the associated gene expression profiles and biological functions. Their results indicated that *ARID2* knockout contributed to disrupting the nucleotide excision repair (NER) process by suppressing the recruitment of complementation group G (XPG), which resulted in compromised DNA damage and repair, susceptibility to carcinogens and potential hypermutation, thus revealing a novel therapeutic target in HCCs harbouring *ARID2* mutations.^100^, ^101^ Moreover, hepatitis B virus X protein (HBx) expression was also shown to be negatively associated with *ARID2* expression in HCC tissues.^102^, ^103^ Mechanistically, the promoter region of the *ARID2* gene which is inhibited by HBx is located at ‐1040 to ‐601 bp and it contains potential atonal homolog 1 (*ATOH1*) binding elements. The ectopic expression or mutation of *ATOH1* binding sites in the *ARID2* promoter partially abolishes HBx‐triggered *ARID2* transcriptional repression. The ATOH1‐mediated deregulation of *ARID2* by HBx may be involved in the development of HBV‐related HCC. These results suggest that mutations in *ARID2* which are associated with HBx may result in a defect in the SWI/SNF complexes, thereby not only leading to abnormal gene expression but also greatly aggravating DNA damage. Eventually, this may trigger carcinogenesis due to the diminished response of the repair mechanisms.

### 3.2 *BAF60a* and *SMARCC1*/*BAF155*

Hepatic BAF60a, which serves as a linker between the SWI/SNF core complex and those TFs regulating target gene expression, senses and integrates environmental factors (such as starvation, circadian clock and dietary fat consumption) into the transcriptional reprogramming and metabolic adaptations.^104^ The gene programme governing β‐oxidation of FAs can be bridged to the SWI/SNF complexes by BAF60a, which indicates that this SWI/SNF chromatin remodelling factor may be implicated in lipid metabolic reprogramming, an evolving hallmark of HCC. In addition, Chen et al demonstrated that over‐expression of *BAF155* enhanced the transcriptional transactivation function of HBx, activated proto‐oncogene expression and inhibited hepatoma cell clonogenicity. The authors also suggest that BAF155 plays important roles in ubiquitin‐independent degradation of HBx, which might be related to the pathogenesis and carcinogenesis of HBV‐associated HCC.^105^ Studies focused on understanding the contributions of BAF60a and BAF155 to HCC, and how these factors interact with other SWI/SNF‐related subunits should be conducted.

## 4 CONTRIBUTIONS TO ONCOGENESIS

Although the potential mechanisms through which SWI/SNF complexes inhibit or promote HCC have been described in the different subsections above, insight into the specific mechanisms underlying tumour suppression or promotion and the reasons for the differing cancer spectrum associated with each subunit is still in its infancy. In addition to a role in transcriptional regulation, several avenues of research have linked the SWI/SNF complex to DNA repair including nucleotide excision repair, double‐strand break repair and DNA decatenation.^106^, ^107^, ^108^ This begs the question as to whether the tumour suppressor activity of the complex arises via a role in controlling transcriptional programmes or whether it is derived from a role for the complex in protecting genome integrity. Mutations in some subunits, such as those in ARID2 mentioned earlier, may cause hepatocarcinogenesis associated with DNA damage and repair, while deletions in other subunits, such as SMARCB1, may not cause cancer via defects in DNA repair but rather due to epigenetic alterations such as disruption of chromatin‐based contributions to the control of cell fate.^58^ Of particular interest is the finding that mutations in more than one SWI/SNF subunit gene can occur in primary tumours, perhaps reflecting both haploinsufficiency and compound heterozygous effects.^109^, ^110^ Whether the mutations of the SWI/SNF subunit genes drive or repress tumours, especially HCC, via an epigenetic mechanism or a genetic mechanism remains to be determined.

## 5 TARGETING TUMOURS WITH BAF/PBAF DEFICIENCIES

One anticancer drug discovery strategy, which reveals great promise in targeting cancer cells with genetic mutations, is the exploitation of synthetic lethality.^111^ SMARCA4 and SMARCA2 function as mutually exclusive catalytic subunits of the SWI/SNF complex, and Hoffman et al^112^ demonstrated that depletion of *SMARCA2* in *SMARCA4*‐deficient cancer cells led to cell cycle arrest and senescence. Recent studies also reported that inhibition of enhancer of zeste homolog 2 (EZH2), the catalytic subunit of PRC2, caused synthetic lethality in *ARID1A*‐mutated cancers.^113^ Unfortunately, epigenetic drugs (such as EZH2 antagonists) did not exhibit synthetic lethality in ARID2‐disrupted HCC, while exposure of ultraviolet irradiation and chemical compounds regulating the DNA repair system will be the effective treatment.^100^ Additionally, the loss of function of BAF/PBAF subunits may lead to increased Polycomb activity and inhibition of Polycomb chromatin silencing may therefore be beneficial for patients with HCC bearing BAF/PBAF deficiencies.^58^ Nevertheless, the effectiveness of these approaches may depend greatly on the downstream consequences of BAF/PBAF dysfunction within each cell type.

## 6 RELATIONSHIP OF THE SWI/SNF COMPLEXES WITH IMMUNITY

### 6.1 Roles of the SWI/SNF complexes in modulating the immune system

An accumulating body of literature has described the effect of the mutated SWI/SNF complexes on various immune cells at various levels. Holley et al^114^ reported that SMARCA4 promoted B‐cell activation, as characterized by the expression of inducible genes, resulting in accelerated cell proliferation and improved immune function. Another study suggested that specifically over‐expressed *SWI3*‐related gene 3 (*SRG3*) utilizes the CD2 promoter to allow cells to up‐regulate the differentiation of CD4^+^ T cells into Th1/Th17 cells, whereas the ubiquitous over‐expression of *SRG3* using the β‐actin promoter facilitates Th2 differentiation and suppressed Th1 and Th17 differentiation.^115^ Furthermore, *SRG3* over‐expression not only reduces the production of pro‐inflammatory cytokines by dendritic cells (DCs), but also shifts macrophages from the inducible nitric oxide synthase (iNOS)‐expressing M1 phenotype to the arginase‐1‐expressing M2 phenotype. The above‐mentioned evidence supports the development of a novel therapeutic strategy involving the modulation of *SRG3* expression to induce M2 and Th2 polarization, thereby inhibiting inflammatory immune responses, which has the potential to be employed in the treatment of immune diseases and tumours. In addition, Thomas et al reported that T cell‐specific *SMARCA4*‐deficient mice exhibited profound thymic abnormalities, CD4 derepression at the double negative (DN) (CD4^−^ CD8^−^) stage and a developmental block hindering the transition from DN to double positive (CD4^+^ CD8^+^) stage. It was therefore speculated that SMARCA4 exerts a vital role in genes important for T‐cell differentiation.^116^ Of particular interest is research suggesting that in contrast to the high incidence of CD8^+^ lymphomas observed through conditional inactivation of SNF5 (another SWI/SNF‐related complex member),^117^ the loss of SMARCA4 function did not lead to deregulated cell proliferation and T cell‐derived tumour formation in mutant animals that were over 18 months old. This evidence supports the notion that the putative tumour suppressor function associated with SMARCA4 loss‐of‐function mutations in human and mouse neoplasms is tissue and cell type‐dependent and further does not predispose cells of the mature T‐cell lineage to oncogenic transformation. With regard to regulatory T cells (Tregs), Chyaiyachati et al^118^ provided evidence that SMARCA4 bound to the CXC chemokine receptor 3 (CXCR3) and chemokine receptor 2 (CCR2) loci in Tregs, whereas the deletion of *SMARCA4* impaired the activation of Tregs in response to self‐antigen and T‐cell receptor (TCR) signalling. The authors demonstrated that SMARCA4 promoted Treg activation and immune tolerance, which overweighed its pro‐inflammatory role in conventional T cells, and while *SMARCA4* knock‐down Tregs were partially activated by persistent and severe inflammation, this activation was insufficient to stop the inflammation. As mentioned above, SMARCA4 is more inclined to accelerate tumour development in the context of HCC and we speculate that this may be partly due to its effect in promoting immune tolerance.

**FIGURE 4:**
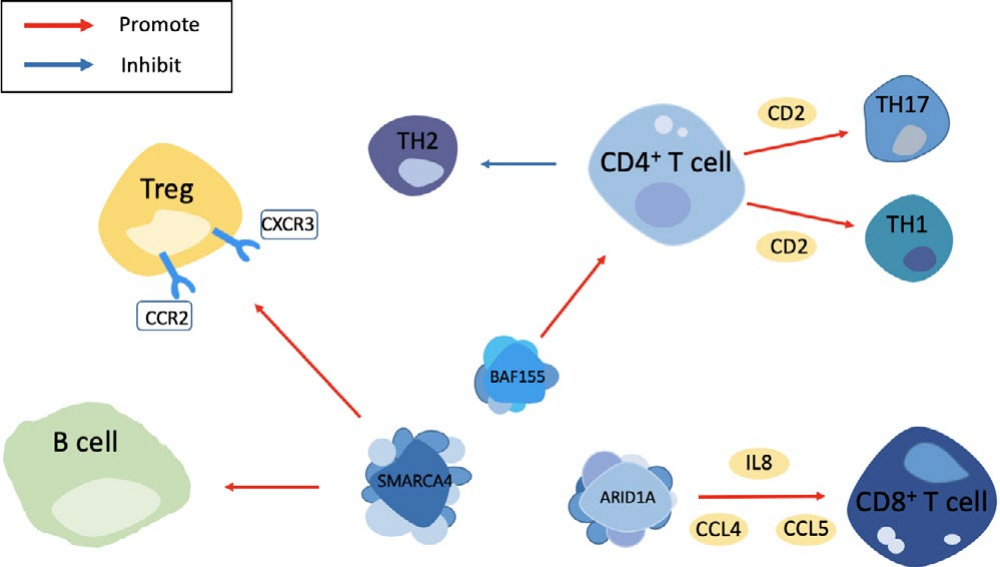
The effect of SWI/SNF complex on the proliferation and differentiation of several immune cells. See the main text for detailed description of the relationship shown. CCR2, chemokine receptor 2; CXCL3, CXC chemokine receptor 3; Treg, regulatory T cells; TH, T helper; CCL4, C‐C motif chemokine 4‐like; IL‐8, interleukin‐8

With respect to the other subunits, RNAi screening revealed that *ARID1A* knock‐down promotes the resistance of Jurkat leukaemia cells to Fas (CD95)‐mediated apoptosis, a cell‐killing mechanism adopted by T cells and NK cells.^119^, ^120^ The association between ARID1A mutations and cancer subtypes with profound lymphocytic infiltration raises the possibility that ARID1A mutations may boost the ability of cancer cells to escape from immune surveillance. In addition, a study on ovarian cancer using differential expression analysis discovered that *SMARCE1* mRNA levels are closely correlated with the number of intra‐tumoral CD8^+^ cells.^121^ Specifically, the forced over‐expression of *SMARCE1* in ovarian cancer cells induces the secretion of IL8, MIP1b and CCL5 chemokines in the supernatant and triggers the chemotaxis of CD8 + lymphocytes in a cell culture assay. In short, the SWI/SNF complexes exert their own influence in the growth, development and recruitment of immune cells, such as T cells and B cells (Figure 4). Indeed, the alteration of chromatin dynamics accounts for a potential mechanism that induces target gene expression in the case of immune cell activation. Therefore, it is important to explore the roles of the SWI/SNF complexes in modulating the immune system, as this may facilitate the formulation of immunotherapies that can achieve remarkable results.

### 6.2 The SWI/SNF complexes are involved in enhancing the transcription of immunostimulatory interferon‐stimulated genes (ISGs)

**FIGURE 5:**
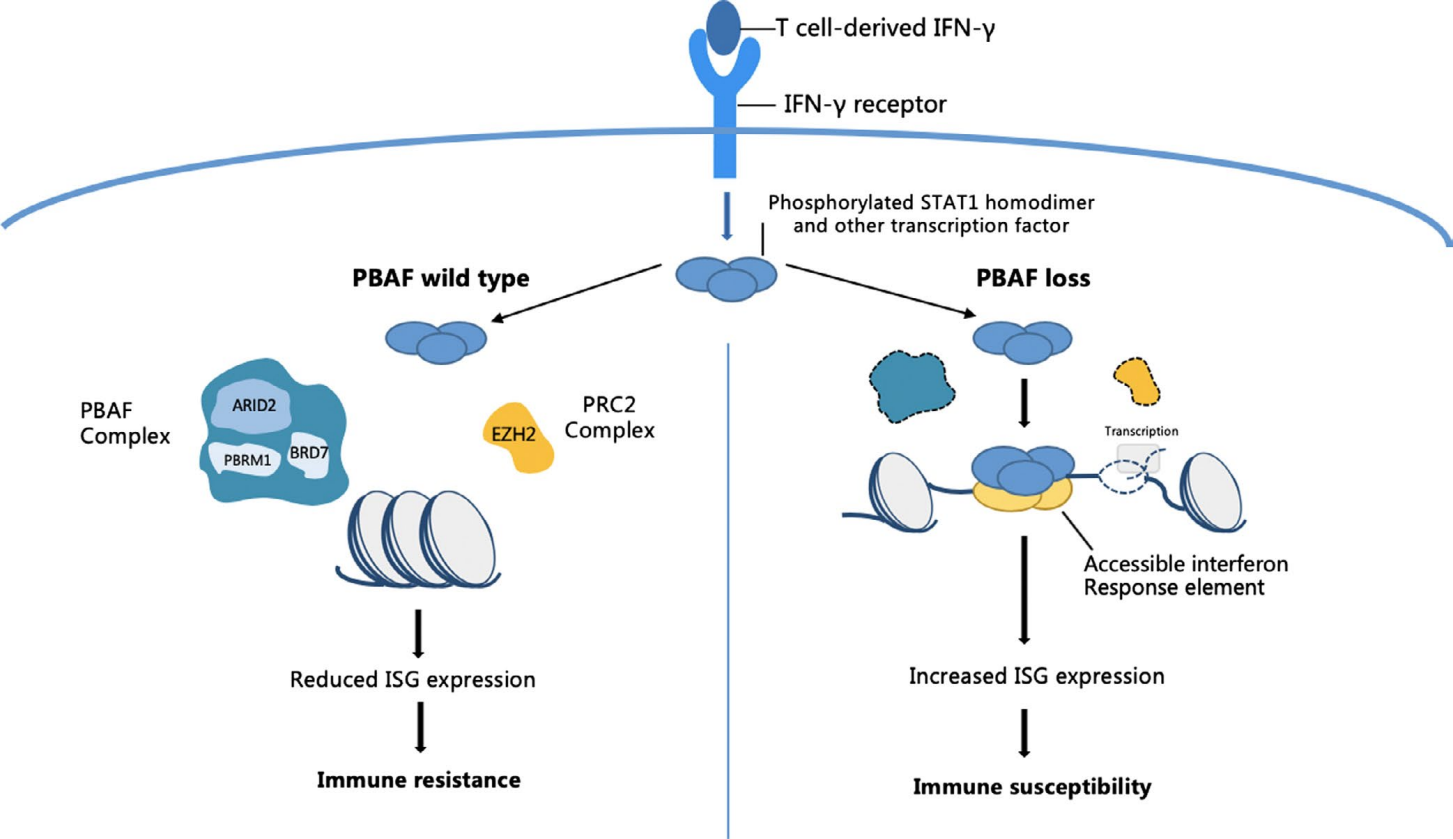
Loss of PBAF could improve the expression of ISGs. Loss of PBAF is proposed to alter chromatin structure so that IFN‐g response elements in ISG promoters are more accessible to transcription factors, increasing their expression. When PBAF is intact, it might cooperate with EZH2 to modify chromatin and reduce the accessibility to IFN‐g response elements. PBAF, polybromo BAF; ISGs, interferon‐stimulated genes

Interferons (IFNs) are a group of cytokines with antitumour effects which are mediated by the activation of Janus kinases (JAKs) and STATs.^122^, ^123^ The lack of an IFN‐γ signature is suggested to be correlated with resistance to immunotherapy.^124^ In addition to enhancing tumour cell immunogenicity through up‐regulating the expression of tumour antigen processing and presentation, ISGs also promote programmed cell death and anti‐proliferative effects.^125^ Moreover, STAT proteins that bind to ISG promoters are regulated by the multi‐protein complexes responsible for chromatin remodelling, including the SWI/SNF complexes, so as to control DNA accessibility. According to previous studies, the SWI/SNF complexes are implicated in enhancing ISG transcription. Patibandla et al reported the increased expression of C‑C motif CCL5 and CXCL10, the two cGAS‐dependent IFN‐stimulated genes, in cell lines with *SMARCA4* knock‐down. The authors further demonstrated that the loss of SMARCA4 might have a certain bearing on the activation of the innate immune response in SCCOHT.^126^ Furthermore, Pan et al identified three PBAF‐specific genes (namely *BRD7*, *ARID2* and *PBRM1*) by conducting a CRISPR screen in the ICI‐resistant mouse melanoma cell line. Importantly, these three genes are involved in antigen presentation and IFN‐γ signalling, which are important for immune sensitivity and imply a role in conferring resistance to T‐cell attack.^127^ ICI‐resistant melanoma cells are more sensitive to IFN‐γ, leading to the increased secretion of cytokines to promote antitumour immunity. In addition, *Pbrm1*‐deficient melanomas in mice also show greater T‐cell infiltration and sensitivity to the anti‐PD‐1 combined with anti‐CTLA‐4 ICIs, which is consistent with the above reports. Recently, researchers have paid particular attention to the mechanism by which the loss of function of PBAF promotes ISG transcription. Typically, Polycomb repressive complex 2 (*PRC2*), the epigenetic silencer, is over‐expressed in cancer cells and mediates the repression of multiple IFN‐γ‐stimulated genes. PBRM1 also cooperates with the PRC2 subunit, EZH2, to promote ISG silencing, which may explain the association of PBAF loss with the induction of ISG expression (Figure 5).^128^, ^129^ In the light of the tumour suppressor effects of PBAF, it is interesting that the inactivating mutations in PBAF subunits sensitize cancer cells to T cell–mediated destruction, which suggests a trade‐off between the concurrent enhanced tumorigenicity and tumour suppressor immunogenicity. Taken together, PBAF deficiency may serve as a biomarker of treatment response, thereby assisting in selecting appropriate treatments for patients. It has recently been suggested that EZH2 inhibition enhances ICI efficacy in melanoma mouse models, and together, these findings pave the way for clinical trials that integrate small‐molecule inhibitors of the chromatin remodelling pathways with ICIs as a novel synergistic treatment combination.

### 6.3 Mutated SWI/SNF complexes shape the response of immunotherapy in different tumours

As suggested in recent literature, compared with SWI/SNF wild‐type colorectal cancer (CRC), tumours harbouring SWI/SNF gene mutations show dramatically higher rates of microsatellite instability (MSI)‐high, tumour mutational burden (TMB)‐high and PD‐L1 positivity, exhibiting a close correlation with the immune profile.^130^ It has also been demonstrated that this subgroup of CRCs may possess a higher capacity to respond to monoclonal antibodies targeting PD‐1 and PD‐L1, further elucidating the relationship between the mutated SWI/SNF complexes and immunotherapy. On the other hand, mutation or low expression of the chromatin remodelling genes, including *SMARCA2* and *PBRM1*, is reported to be associated with larger neoantigen burdens and a greater amount of activated CD8^+^ T‐cell infiltration in human non–small‐cell lung carcinoma.^131^, ^132^ Tumours with low *PBRM1* expression also show a favourable prognosis and are connected to the increase in cytotoxic lymphocytes in their TME.^133^ Furthermore, *ARID1A* deficiency is correlated with a microsatellite instability genomic signature, a predominant C>T mutation pattern and an increased mutation load across multiple human cancer types.^134^ Shen et al showed that tumours with an *ARID1A*‐deficient ovarian cancer cell line origin in syngeneic mice displayed increased mutation load, enhanced levels of tumour‐infiltrating lymphocytes and up‐regulated *PD‐L1* expression. In contrast, treatment with anti‐PD‐L1 antibody reduced the tumour burden and prolonged the survival of mice bearing an *Arid1a* deficiency but not *ARID1A* wild‐type ovarian tumours. Taken together, such findings reflect that the loss of ARID1A may cooperate with immune checkpoint blockade therapy. Outlining the role of ARID1A in immune escape may provide more insights into the efficacy of immunotherapy‐based approaches. In a study conducted by Miao et al,^135^ the authors independently analysed genes related to the SWI/SNF complexes and found that most patients harbouring truncating mutations of *PBRM1* and *ARID2* benefited from ICI treatments. Furthermore, patients with truncating mutations of *ARID1B*, *DPF2* and *SMARCA2* (exclusively belonging to BAF), together with *SMARCA4*, were also more inclined to be relieved by ICI treatments. A recent study also suggested that ICIs may be used as favourable candidates for treating *ARID2*‐mutated cancers displaying the hypermutator phenotype, including HCC.^100^ ICIs have demonstrated great survival benefits for HCC.^136^ Future studies should shed light on the therapeutic effect of ICIs on patients with SWI/SNF‐mutated HCC. In summary, a body of evidence has accumulated indicating that tumour patients with mutations in the SWI/SNF complexes may show a response to ICI treatments, which also suggests new opportunities for tumour immunotherapy.

### 6.4 Correlation of the SWI/SNF complexes with immunity in HCC

To date, only a few studies have reported the influence of the mutated SWI/SNF complexes on the immune microenvironment, as well as synergistic effects with immunotherapy among HCC patients. As mentioned earlier, Fang et al demonstrated that steatohepatitis and HCC development were observed in hepatocyte‐specific *Arid1a* knockout mice, which was accompanied by infiltration of innate immune cells (including F4/80^+^ macrophages and CD11c^+^ neutrophil cells) into the liver parenchyma and increased levels of tumour necrosis factor (TNF)‐α and interleukin (IL)‐6, as well as activation of the STAT3 and NF‐κB pathways. These results strongly suggested that the mutated SWI/SNF complexes might affect the immune microenvironment of HCC. On the other hand, when mice were fed with a methionine and choline deficiency (MCD) diet, liver injury and hepatic inflammation were mitigated in the hepatocyte‐specific *Smarca4* knockout mice compared with their wild‐type littermates. Moreover, the synthesis of pro‐inflammatory mediators was also down‐regulated in primary hepatocytes isolated from the *Smarca4*‐deficient mice relative to the WT mice, resulting in reduced macrophage chemotaxis.^137^ The mechanism by which SMARCA4 contributes to the transcription of pro‐inflammatory mediators is possibly through the regulation of the interaction between NF‐κB and its co‐factor myocardin‐related transcription factor A (MRTF‐A). Non‐alcoholic steatohepatitis (NASH) has been recognized as a major catalyst of HCC, and the hepatocyte‐specific deletion of *SMARCA4* relieves MCD‐induced NASH in mice.^138^, ^139^, ^140^ Nonetheless, more in‐depth research is warranted to shed more light on how the regulation of aberrant SWI/SNF complexes affects the immune microenvironment of HCC and to lay a foundation for the novel immunotherapies.

## 7 CONCLUSIONS

Studies in animal models and human samples have expanded the body of knowledge regarding the mutation or loss of the SWI/SNF complexes as a significant functional suppressor or promoter of tumorigenesis and tumour progression, especially in HCC. Mounting evidence suggests that the SWI/SNF complexes markedly affect the regulation of ISG transcription, as well as the differentiation, activation and recruitment of several immune cells. In addition, they exhibit a synergistic effect with ICIs in the treatment of diverse tumours, making an argument for the potential roles of such complexes in the immune microenvironment. Nonetheless, multimodal and multi‐agent therapies are required due to the heterogeneity of HCC. Identifying such treatments will gain traction from the high‐throughput screening of target molecules and NGS‐based stratification of patients, to identify and explore novel, effective and more personalized therapeutic approaches.

## CONFLICT OF INTEREST

The authors declare that they have no competing interests.

## AUTHORS' CONTRIBUTIONS

XS, BH and JL created the idea for the review. BH performed the selection of literature, drafted the manuscript and prepared the figures. XY and JL revised the manuscript. All authors read and approved the final manuscript.

## CONSENT FOR PUBLICATION

All authors agree to submit for consideration for publication in the journal.
